# Targeting hyaluronan-mediated motility receptor (HMMR) enhances response to androgen receptor signalling inhibitors in prostate cancer

**DOI:** 10.1038/s41416-023-02406-8

**Published:** 2023-09-06

**Authors:** Josephine A. Hinneh, Joanna L. Gillis, Chui Yan Mah, Swati Irani, Raj K. Shrestha, Natalie K. Ryan, Enomoto Atsushi, Zeyad D. Nassar, David J. Lynn, Luke A. Selth, Masashi Kato, Margaret M. Centenera, Lisa M. Butler

**Affiliations:** 1 South Australian Immunogenomics Cancer Institute, University of Adelaide, Adelaide, SA 5000 Australia; 2 Freemason’s Centre for Male Health and Wellbeing, University of Adelaide, Adelaide, SA 5000 Australia; 3 Precision Cancer Medicine Theme, South Australian Health and Medical Research Institute, Adelaide, SA 5000 Australia; 4 Department of Urology, Nagoya University Graduate School of Medicine, Nagoya, Japan; 5 Adelaide Medical School, University of Adelaide, Adelaide, SA 5005 Australia; 6 College of Medicine and Public Health, Flinders University, Bedford Park, SA 5042 Australia; 7 Flinders Health and Medical Research Institute, Flinders University, Bedford Park, SA 5042 Australia; 8 Department of Pathology, Nagoya University Graduate School of Medicine, Nagoya, Japan

**Keywords:** Prostate cancer, Translational research

## Abstract

**Background:**

Resistance to androgen receptor signalling inhibitors (ARSIs) represents a major clinical challenge in prostate cancer. We previously demonstrated that the ARSI enzalutamide inhibits only a subset of all AR-regulated genes, and hypothesise that the unaffected gene networks represent potential targets for therapeutic intervention. This study identified the hyaluronan-mediated motility receptor (HMMR) as a survival factor in prostate cancer and investigated its potential as a co-target for overcoming resistance to ARSIs.

**Methods:**

RNA-seq, RT-qPCR and Western Blot were used to evaluate the regulation of HMMR by AR and ARSIs. HMMR inhibition was achieved via siRNA knockdown or pharmacological inhibition using 4-methylumbelliferone (4-MU) in prostate cancer cell lines, a mouse xenograft model and patient-derived explants (PDEs).

**Results:**

HMMR was an AR-regulated factor that was unaffected by ARSIs. Genetic (siRNA) or pharmacological (4-MU) inhibition of HMMR significantly suppressed growth and induced apoptosis in hormone-sensitive and enzalutamide-resistant models of prostate cancer. Mechanistically, 4-MU inhibited AR nuclear translocation, AR protein expression and subsequent downstream AR signalling. 4-MU enhanced the growth-suppressive effects of 3 different ARSIs in vitro and, in combination with enzalutamide, restricted proliferation of prostate cancer cells in vivo and in PDEs.

**Conclusion:**

Co-targeting HMMR and AR represents an effective strategy for improving response to ARSIs.

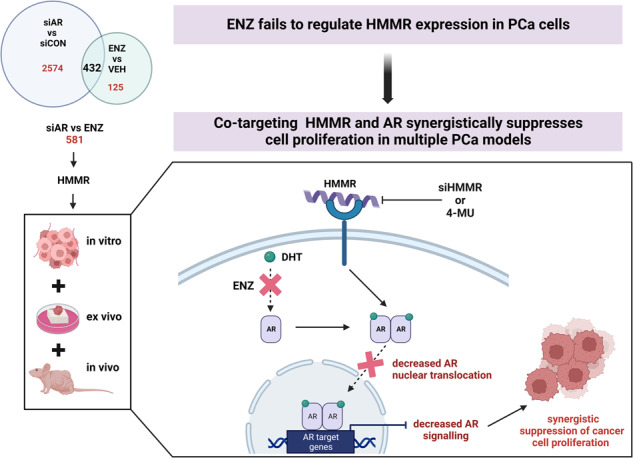

## Introduction

Prostate cancer (PCa) is the most frequently diagnosed cancer and the second leading cause of cancer-related death in men in the developed world [1]. A hallmark of PCa is its dependence on androgen signalling, hence the androgen receptor (AR) is the primary therapeutic target for advanced disease [2, 3]. While inhibition of AR signalling by androgen deprivation therapy (ADT) has been the mainstay of PCa treatment for almost 80 years, the disease invariably progresses to a lethal form known as castration-resistant prostate cancer (CRPC). A key feature of CRPC is the re-activation of AR signalling, which has underpinned the development of second-generation AR signalling inhibitors (ARSIs) [4]. There are four second-generation ARSIs currently approved by the Food and Drug Administration (FDA); the androgen biosynthesis inhibitor abiraterone acetate [5], and the AR antagonists enzalutamide [6, 7], apalutamide [8] and darolutamide [9]. While ARSI use improves survival, resistance to these agents has become one of the most important challenges in the clinical management of PCa. Intrinsic resistance to enzalutamide, for example, is observed in up to 50% of patients treated, and those who initially respond will generally acquire resistance within a period of months [10]. Mechanistically, resistance to ARSIs is largely attributed to aberrations in *AR*, such as mutations, amplification and splicing variants [11], highlighting the continued dependence of CRPC on AR signalling.

For a decade, combination approaches to target AR signalling in PCa have been postulated as essential to achieve long-term disease control [12], but combination strategies have still not been optimised. There are a multitude of PCa clinical trials investigating combinations of approved agents, including ADT, ARSIs, radiotherapy, chemotherapy, immunotherapy and small molecular inhibitors. Indeed, triplet therapy that combines ADT, chemotherapy and an ARSI may soon become the new gold standard for hormone-sensitive metastatic PCa based on recent trial results [13]. While this and other combination strategies have been successful in extending survival, they have largely been based on existing and available agents. The aim of our study was to rationally design a novel combination treatment strategy for PCa that more effectively inhibits AR signalling and PCa cell survival. Previously we reported that the AR antagonist enzalutamide does not effectively target the entire repertoire of genes regulated by the AR in PCa cells, and presented evidence that those remaining networks provide fuel for survival [14]. Herein, we investigate the function of one such gene, hyaluronan-mediated motility receptor (*HMMR*), and the therapeutic potential of targeting HMMR to sensitise tumour cells to enzalutamide.

## Materials and Methods

### Cells lines and reagents

VCaP, LNCaP, 22Rv1 and PC3 human prostate carcinoma cells were purchased from the American Type Culture Collection (ATCC, MD, USA). LNCaP-derived V16D (castration-resistant, enzalutamide-sensitive) and MR49F (castration-resistant, enzalutamide-resistant) human PCa cells were a kind gift from Prof. Amina Zoubeidi (Vancouver Prostate Centre, Vancouver, Canada) [15]. All cell lines were verified using short tandem repeat profiling in 2022 by ATCC or CellBank Australia. LNCaP, 22RV1, MR49F and V16D cells were maintained in RPMI-1640 supplemented with 10% FBS; the media for growth of MR49F cells was additionally supplemented with 10 µM enzalutamide. VCaP cells were maintained in Dulbecco’s Modified Eagle’s Medium containing 10% FBS, 1% sodium pyruvate, 1% MEM non–essential amino acids, and 0.1 nM 5α-dihydrotestosterone (DHT).

All chemicals, reagents and 4-methlumbelliferone were purchased from Sigma Aldrich (St Louis, MO, USA). Enzalutamide was obtained from Selleck Chemicals (Houston, TX, USA); apalutamide (ARN-509), darolutamide (ODM-201) from Sapphire Bioscience (Redfern, NSW, AUS) and Hyaluronic acid from Contipro (Dolní Dobrouč, Czech Republic). All drugs were dissolved in DMSO except for HA which was dissolved in RNA grade double distilled water.

### Transient transfection

HMMR knockdown was carried out using Dharmacon ON-Target HMMR (3161) SMARTpool 5 nmol siRNA cat#: L-010409-00-0005 and control cells were transfected with Dharmacon ON-Target Plus siRNA cat#: D-001810-01-20 siRNA at a concentration of 12.5 nM for 6 well plates (5 × 10^5^ cells), 5 nM for 24 and 96 well plates (3.5 × 10^4^ cells and 3.5 × 10^3^ cells respectively). The cells were reverse transfected using lipofectamine RNAiMAX (Life technologies, ThermoFisher Scientific, Scornsby, VIC, AUS) according to the manufacturer’s protocol. AR knockdown was achieved as previously described [14]. The following AR siRNAs were used: AR (Silencer Select #4390824/5; s1538 (siAR2), s1539 (siAR1) (Ambion; ThermoFisher Scientific), and Negative Control 2 #AM4637 (Ambion; ThermoFisher Scientific).

### Cell viability and apoptosis assays

Cells were seeded in triplicate in 24 well plates at densities of 3.5 × 10^4^ for LNCaP, 3 × 10^4^ for MR49F and 2.5 × 10^4^ for V16D. Cell viability was determined by manual counting using Trypan blue exclusion, as described previously [16]. Alternatively, cell viability was assessed by CyQuant^TM^ Cell Proliferation Assay (ThermoFisher Scientific), according to the manufacturer’s instructions.

The effect of siRNA or drug treatment on apoptosis was carried out by seeding 5 × 10^4^ cells/well in triplicate. Cells were collected three days post-treatment or knockdown and stained with Annexin V PE (BD Pharmagen^TM^, CA, USA) and 1 mM 7-Aminoactiomycin D (Thermo Fisher Scientific) and analysed using a BD LSRFortessa X20 Flow Cytometer.

### Quantification of combination index

The combination index (CI) was determined using Compusyn software (Compusyn Inc. Paramus, NJ, USA) based on the Chau-Talalay theorem [17]. The quantitative value of the CI is defined as follows: CI = 1: additive effect, CI > 1: antagonism, CI < 1: synergy.

### Colony formation assay

Cells were seeded at 500 cells/well in 2 mL RPMI + 10% FBS and incubated at 37 °C for 2 weeks with the indicated treatments, fixed with 4% paraformaldehyde and stained with 1% crystal violet to identify colonies. Colony-forming efficiency was determined by counting colonies greater than 50 cells for each well.

### Quantitative real-time polymerase chain reaction (RT-qPCR)

RNA (1 µg) was reverse transcribed using iScript cDNA synthesis kit (Bio-Rad, CA, USA), according to the manufacturer’s instructions. RT-qPCR was performed on 1:10 diluted cDNA using SYBR green and CFX384 Real-Time System (Bio-Rad) for 40 cycles. Gene expressions are presented relative to *GUSB* and *L19* as determined by GeNorm [18]. All primers were purchased from Sigma Aldrich with primer sequences listed in Supplementary Table 1.

### Immunoblotting

Whole-cell lysates were collected in RIPA lysis buffer supplemented with cOmplete ULTRA protease and phosphatase inhibitor (Cell Signalling Technology (CST), Danvers, MA, USA) and immunoblotting was performed as previously described [19]. Primary and secondary antibodies used in this study are enlisted in Supplementary Table 2.

### Visualisation of pCMV-tagged AR protein

PC3 cells at a density of 4 × 10^4^ cells/well were reverse transfected with 1 µg pCMV-AR in an 8-well Lab-Tek II chamber slide (ThermoFisher Scientific) using lipofectamine 2000 (ThermoFisher Scientific) according to the manufacturer’s protocol and allowed to adhere for 4 h. An overlay of drug 4-MU or docetaxel was added and incubated for 24 h. Cells were then treated with 1 nM DHT or vehicle for 4 h and fixed with 4% paraformaldehyde. Fixed cells were washed in PBS and incubated with an antibody against AR (AR-N20) overnight. AR was visualised using Alexa-Fluor 488 goat anti-rabbit IgG (ThermoFisher Scientific). Images were obtained using Leica TCS SP8X/MP confocal microscope (Leica Microsystem, Wetzler, Germany). Nucleoplasmic translocation was quantified using FIJI software (ImageJ: http://fiji.sc/Fiji; version 1.52 p) [20].

### Subcellular fractionation of prostate cancer cells

LNCaP cells at 80% confluency were androgen starved for three days and treated with PRF-RPMI containing vehicle, 0.4 mM 4-MU for 24 h followed by 10 nM DHT or vehicle treatment for 4 h. Cells were trypsinised, 200 µL was collected as whole cell lysate and the remaining cell suspension centrifuged at 1500 rpm for 5 mins to pellet the cells. The cell pellet was washed in PBS and lysed in 500 µL lysis buffer 1 (10 mM Tris HCl (pH 7.9), 0.34 M sucrose, 3 mM CaCl_2_, 2 mM magnesium acetate, 0.1 mM EDTA, 1 mM DTT, 0.5% nonidet P-40 substitute) containing cOmplete ULTRA protease (CST, Danvers, MA, USA) and centrifuged at 3500 x *g* for 5 min. The supernatant was clarified by spinning at 20,000 x g for 15 mins and saved as the cytoplasmic fraction. The cell pellet was then washed with 300 µl lysis buffer 1 and pelleted by centrifugation at 3500 x g for 5 mins. The cell pellet was resuspended in 225 µl lysis buffer 2 (20 mM HEPES (pH 7.9), 3 mM EDTA, 10% glycerol, 150 mM potassium acetate, 1.5 mM MgCl_2_, 1 mM DTT, 0.1% Nonident P-40 substitute) containing cOmplete ULTRA protease and phosphatase inhibitor (CST, Danvers, MA, USA) and centrifuged at 15000 x g for 30 mins. The supernatant was saved as the nuclear fraction. The cell pellet was resuspended in 300 µl PBS and sonicated by BioRuptor sonication system (Diagenode, Belgium) at high intensity 4 × 30 s with 1 min rest in between. The sonicated solution was used as the chromatin fraction. Equivalent volumes of cytoplasmic and nuclear plus chromatin fractions were immunoblotted for AR(AR-N20). Cytoplasmic marker GAPDH and nuclear marker H3 were used as sub-fractionation markers.

### In vivo studies

All animal experiments were approved by The University of Adelaide and Nagoya University (ethics approval numbers: M-2020-014 and 31460 respectively) ethics committees. Balb/cSlc (*nu/nu*) 6 weeks old male nude mice were subcutaneously injected with 1:1 PBS/matrigel suspension containing 2 × 10^6^ V16D cells. Once tumours became palpable, mice were assigned randomly to one of the 4 groups (Vehicle, 225 mg/kg 4-MU, 10 mg/kg ENZ and 225 mg/kg 4-MU + 10 mg/kg ENZ). For 5 weeks, mice were fed with a powdered diet supplemented with vehicle or 4-MU, ENZ or 4-MU + ENZ. Tumour dimensions were measured every second-day using callipers and volumes were calculated using the formula (width^2^ x length x 0.5236). Mice were culled when tumour volume reached 1000 mm^3^ or at the end of 5 weeks of treatment. Tumours were resected and formalin-fixed for immunohistochemistry (IHC) analysis and plasma samples were collected at sacrifice.

### Ex vivo studies

PCa specimens were obtained with informed written consent through St Andrew’s Hospital (Adelaide, Australia) or the Nagoya University Hospital (Nagoya, Japan) from men who underwent radical prostatectomy. Experiments were approved by The University of Adelaide (approval: H-2012-016) and Nagoya University (approval: 2020-01-17) human research ethics committees. Patient-derived explants (PDEs) were generated from 1–2 mm^3^ tissues cultured on gelatin sponges pre-soaked in media as described previously [21]. PDEs were treated with DMSO or 4-MU alone or in combination with enzalutamide, as indicated for each experiment for 48 h. Tissues were formalin-fixed and paraffin embedded for analysis by IHC. AR knockdown in PDEs was performed as previously described [22].

### Immunohistochemistry (IHC)

Tissue sections (2 µm) on Superfrost plus slides were dewaxed and quenched for peroxidase activity using 3% H_2_O_2_ for 15 mins. Target antigens were stained using antibodies detailed in Supplementary Table 2. For animal studies, whole slide images of Ki67 stained sections were obtained using the visual slide system VS120 (Olympus, Tokyo, Japan) and images were quantified using semi-automated TissuemorphDP^TM^ software (Visiopharm, Hørsholm, Denmark), according to the manufacturer’s instructions. For patient-derived explants, Ki67 stained slides were imaged and quantified in a blinded manner, as previously described [21].

### Statistical analysis

Statistical significance was determined using GraphPad Prism version 9 with Student’s t-test, one-way or two-way ANOVA (with Tukey or Dunnett’s post hoc tests), as specified in the figure legends. A *p*-value ≤ 0.05 was considered statistically significant.

## Results

### Discovery of HMMR as a potential pathway to ARSI resistance

**Fig. 1:**
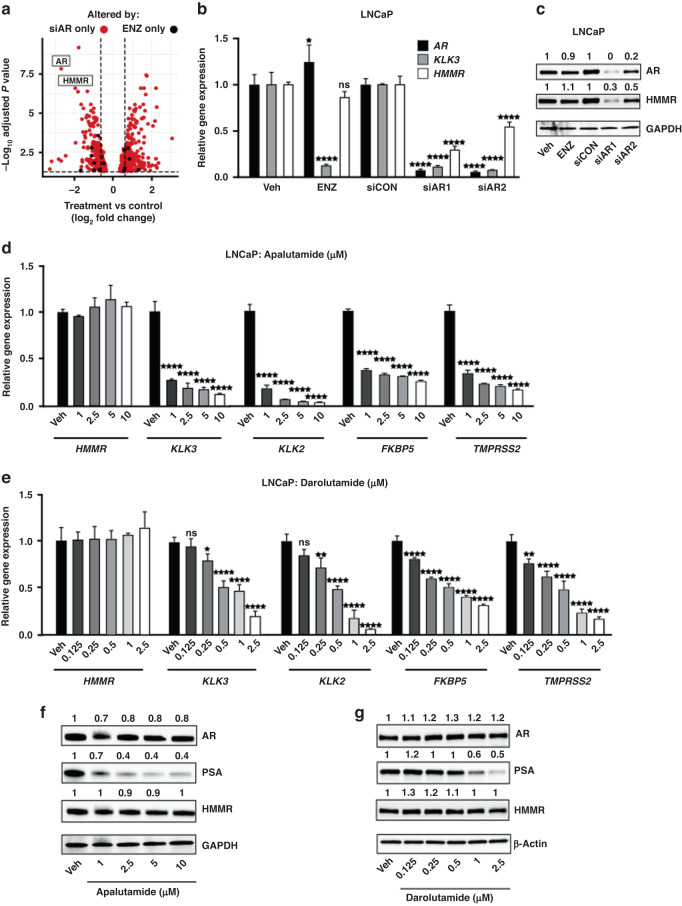
HMMR is suppressed by AR knockdown but not by treatment with ARSIs. **a** Volcano plot of differentially expressed genes affected by either siAR versus siCON (red; *n* = 599 genes), or Veh versus ENZ (black; *n* = 23 genes). **b** Validation of *HMMR* expression in response to siAR or 1 µM ENZ by RT-qPCR. Gene expression was normalised to *GUSB* and *L19*. Data is presented as mean ± SD of 3 biological replicates and are representative of 3 independent experiments. Controls (Veh and siCON) were set to 1 and data statistically evaluated using two-way ANOVA with Tukey’s multiple comparison test (treatment *vs* control; ****p < 0.0001). **c** AR and HMMR protein expression in response to siAR or 1 µM ENZ by Western Blot. GAPDH was used as a loading control.*KLK3*, *KLK2*, *FKBP5* and *TMPRSS2* expression in response to treatment with increasing dose of ARSIs apalutamide (**d**) or darolutamide (**e**) for 24 h, by RT-qPCR. Gene expression was normalised to GUSB and L19. Data are presented as mean ± SD of 3 biological replicates and are representative of three independent experiments. Control (Veh) was set to 1 and data was statistically evaluated using one-way ANOVA with Dunnett’s multiple comparison test. (*p < 0.05, **p < 0.01, ****p < 0.0001). AR, PSA and HMMR protein expression in response to treatment with ARSIs apalutamide (**f**) or darolutamide (**g**) for 24 h, by Western Blot. Numerals above each lane represent densitometric analysis of each protein relative to loading controls GAPDH (**f**) or β-Actin (**g**).

Using an unbiased transcriptomics approach previously reported by our team [14], *HMMR* was identified as one of the most differentially regulated genes by AR knockdown that remains unaffected by enzalutamide treatment (Fig. 1a). This differential regulation of *HMMR* was validated in LNCaP (Fig. 1b, c), VCaP (Supplementary Figure 1a, b) and V16D (Supplementary Figure 1c, d) PCa cells, where *HMMR* gene and protein expression were unaffected by enzalutamide compared to vehicle treatment, but significantly downregulated by two independent siRNA targeted to AR (siAR) compared to scrambled control siRNA (siCON). In contrast, the canonical AR-regulated gene *KLK3*, was significantly downregulated by both enzalutamide treatment and siAR treatment (Fig. 1b, Supplementary Figure 1a, Supplementary Figure 1c). In support of our findings, stable AR knockdown in PCa cells downregulated *HMMR* in an independent RNA-seq dataset (Supplementary Figure 1e) [23], and in patient-derived explants (PDEs), AR knockdown by siAR-loaded nanoparticles [22] reduced both *AR* and *HMMR* mRNA expression compared to control (Supplementary figure 1f). Similarly, the SU2C dataset confirmed our finding that pharmacological inhibition of AR using ARSIs does not inhibit HMMR gene expression, as HMMR remained unchanged by ARSI treatment in CRPC tumours compared to untreated CRPC (Supplementary Figure 1g) [24]. Given the increasing use of ARSIs in combination with ADT clinically [25], we evaluated HMMR expression in response to enzalutamide under androgen-deprived conditions. To simulate androgen deprivation in vitro, androgen-sensitive LNCaP and VCAP cells and androgen-independent V16D cells were androgen-starved for three days. Upon the addition of enzalutamide, HMMR expression remained unchanged compared to vehicle treatment, whereas KLK3 expression was significantly reduced in the presence of enzalutamide in all three cell lines (Supplementary Figure 1h–j).

Having demonstrated that enzalutamide fails to modulate HMMR expression, in the presence or absence of androgens, we tested whether this was specific to this particular ARSI or a broader feature of second-generation AR antagonists. LNCaP cells were treated with increasing dose of clinical agents apalutamide or darolutamide and HMMR expression was measured by RT-qPCR and Western Blot. No significant change in *HMMR* gene (Fig. 1d, e) or protein (Fig. 1f, g) levels were observed upon either ARSI treatment compared to vehicle-treated cells. In contrast, the expression of androgen-regulated genes *KLK3* (PSA), *KLK2, FKBP5* and *TMPRSS2*, and PSA protein expression were potently and dose-dependently supressed by both agents (Fig. 1d–g). Collectively our results provide evidence that despite being inhibited by AR knockdown, *HMMR* evades pharmacological inhibition of AR with second-generation ARSIs.

### HMMR expression is associated with advanced disease, treatment resistance and poor prognosis

**Fig. 2:**
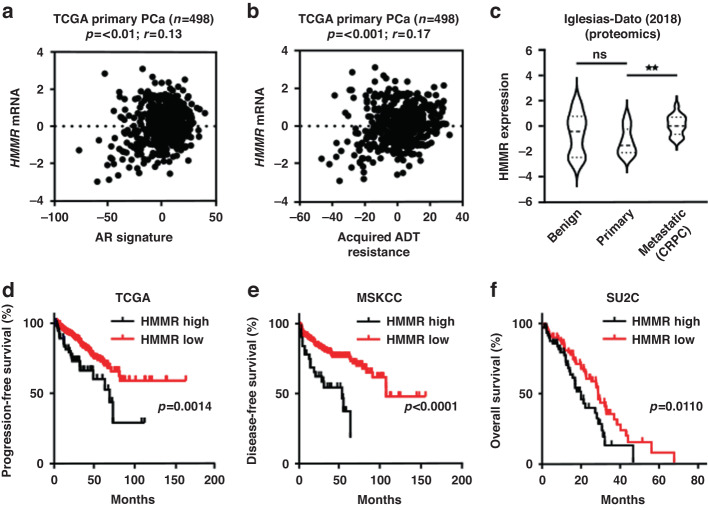
HMMR is clinically significant in prostate cancer. Association between *HMMR* expression levels with an AR signalling signature (**a**) or acquired ADT resistance signature (**b**) from TCGA primary prostate cancer expression data. Scatter plot of the Spearman correlation analysis between HMMR expression and signature genes, where each symbol represents one patient. **c** Violin plot of HMMR protein expression in benign prostate tissues versus primary and metastatic castrate-resistant prostate cancer tissues. Plots show minimum and maximum values (lower and upper lines respectively). Data were analysed using one-way ANOVA with Tukey’s multiple comparison test (**p < 0.01). **d**–**f** HMMR mRNA expression is associated with shorter relapse-free survival in the TCGA (primary prostate cancer) and MSKCC (primary and metastatic CRPC) cohorts, and shorter overall survival rates in the SU2C (metastatic CRPC) cohort.

To determine whether *HMMR* has clinical relevance in PCa, its expression was interrogated in multiple transcriptomic and proteomic datasets. In clinical transcriptomic data from The Cancer Genome Atlas (TCGA) [26], *HMMR* expression was positively correlated with an established AR-regulated gene signature (Fig. 2a), and with acquired ADT resistance (Fig. 2b). This data supports that HMMR is AR-regulated in PCa [27], and that HMMR expression correlates with resistance to androgen deprivation and biochemical recurrence [28]. Increased *HMMR* mRNA expression has been reported in metastatic PCa [29] and CRPC [28] compared to localised disease. We demonstrate the association with metastatic disease is also observed at the protein level, with HMMR overexpressed in metastatic CRPC tumours compared to primary tumours in proteomic data obtained by mass spectrometry [30] (Fig. 2c). Importantly, survival analysis of three independent PCa patient cohorts revealed that high *HMMR* expression is associated with poorer progression-free (Fig. 2d), disease-free (Fig. 2e), and overall survival (Fig. 2f) [26, 31, 32]. In summary, *HMMR* expression is increased in advanced stages of PCa and is associated with treatment resistance and poorer prognosis.

### Targeting HMMR inhibits cell viability and induces cell death of hormone-sensitive and enzalutamide-resistant PCa models

**Fig. 3:**
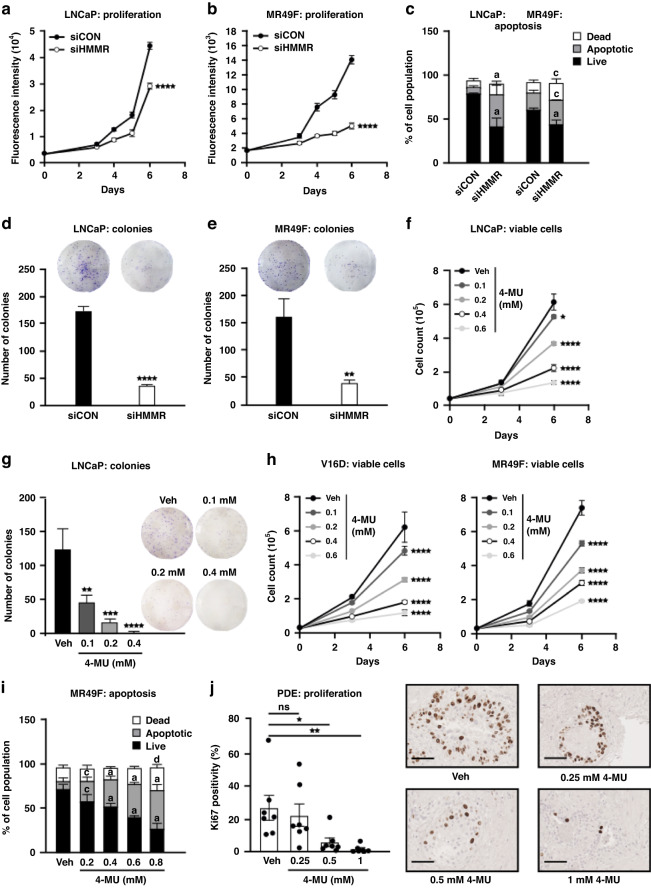
Targeting HMMR inhibits PCa cell growth and survival. Knockdown of HMMR with sMARTpool siRNA (siHMMR) reduced proliferation of LNCaP (**a**) and MR49F (**b**) cells, as determined by CyQuant assay. Cell proliferation was evaluated by measure of fluorescence intensity on days 3-, 4-, 5- and 6 post-transfection. Data are presented as mean ± SD of 5 wells and represent three independent experiments. Data were statistically evaluated using two-way ANOVA with Tukey’s multiple comparison test (****p < 0.0001). **c** HMMR knockdown induced apoptosis of LNCaP and MR49F cells, as determined using flow cytometry-based 7-AAD/Annexin V assays at 3 days post-transfection. Data are presented as mean ± SD of 3 biological replicates and represent three independent experiments. Dead cell proportions upon *HMMR* knockdown were compared to the vehicle using ANOVA and Dunnett’s multiple comparison tests (^a^*p*<0.0001, ^b^*p* < 0.001, ^c^*p* < 0.01). HMMR knockdown with siHMMR inhibits colony formation of LNCaP (**d**) and MR49F (**e**) cells. Formalin-fixed cells were stained with 1% crystal violet then colonies containing >50 cells were manually counted. Data are presented as mean ± SD of 3 wells and represent three independent experiments. Data were statistically analysed using unpaired student’s T-test (**p < 0.01, ****p < 0.0001). (**f**) Pharmacological inhibition of HMMR with 4-MU inhibits LNCaP cell viability. Viable and dead cells were determined by Trypan blue exclusion assay after 3- and 6- days of treatment. Data are presented as mean ± SD of triplicate wells, are representative of two independent experiments, and were analysed using one-way ANOVA with Tukey’s multiple comparison test (*p < 0.05, ****p < 0.0001). **g** 4-MU dose-dependently suppresses colony formation in LNCaP cells. Data are presented as mean ± SD of 3 wells, are representative of two independent experiments, and were analysed using one-way ANOVA with Dunnett’s test. (**p < 0.01, ***p < 0.001, ***p < 0.0001). **h** 4-MU dose-dependently suppresses cell proliferation in V16D and MR49F PCa cells. Cells were counted using the Trypan blue dye exclusion method after 3- and 6- days of treatment. Data are presented as mean ± SD of triplicate wells and represent two independent experiments. Statistical analysis was performed using one-way ANOVA with Turkey’s multiple comparison test. (****p < 0.0001). **i** 4-MU treatment dose-dependently induces apoptosis in MR49F cells three days post-treatment. Data are presented as mean ± SD of 3 wells, represent two independent experiments and were analysed by two-way ANOVA with Turkey’s multiple comparison test (^a^*p* < 0.0001, ^c^*p* < 0.01, ^d^*p* < 0.05. **j** 4-MU inhibits proliferation in patient-derived prostate cancer explants (PDEs). PDEs (*n* = 7) were treated for 48 h with an increasing dose of 4-MU, then paraffin-embedded and formalin-fixed prior to immunohistochemistry (IHC) with proliferation marker Ki67. Digital images were manually counted. Data represents mean ± SEM and were statistically analysed using one-way ANOVA with Dunnett’s test (*p < 0.05, **p < 0.01). Quantification of Ki67 staining on the left and representative IHC images on the right.

Having established that HMMR is not modulated by ARSIs, we investigated its potential as a therapeutic target. Transient downregulation of HMMR using SMARTpool siRNA (siHMMR) markedly inhibited cell proliferation (Fig. 3a, b) and induced apoptosis (Fig. 3c) compared to control siRNA (siCON) in enzalutamide-sensitive LNCaP and enzalutamide-resistant MR49F cells. Additionally, siHMMR suppressed the colony-forming abilities of both LNCaP (Fig. 3d) and MR49F (Fig. 3e) cells, indicative of reduced survival ability. The efficiency of HMMR knockdown with the SMARTpool siRNA was confirmed at gene and protein levels by RT-qPCR and Western Blotting, respectively (Supplementary Figure 2a–c). Having demonstrated that HMMR knockdown regulates PCa cell growth and survival, we sought to inhibit HMMR using a pharmacological agent. While there are no direct inhibitors of HMMR, HA is a well-characterised ligand for HMMR [33], and the HA synthesis inhibitor, 4-methylumbeliferone (4-MU) has previously been reported to suppress HMMR expression [34]. We likewise found that 4-MU suppressed HMMR expression in a range of PCa cell lines (Supplementary Figure 2d–f). 4-MU dose-dependently reduced cell viability (Fig. 3f) and colony formation (Fig. 3g) compared to vehicle treatment in androgen-sensitive LNCaP cells. Importantly, 4-MU was also potent against models of CRPC, including 22Rv1 (Supplementary Figure 2g) and the enzalutamide-sensitive V16D and enzalutamide-resistant MR49F lines (Fig. 3h). In enzalutamide-resistant MR49F cells, 4-MU markedly induced apoptosis, as determined by flow cytometry using 7AAD/Annexin V-PE staining (Fig. 3i). To ensure that the growth inhibitory effects of 4-MU in this study were mediated through HA, we undertook a rescue experiment by co-treating PCa cells with 4-MU and low molecular weight HA (27 kDa). The addition of HA partially rescued the cell proliferative effects of 4-MU in V16D cells (Supplementary Figure 2h), supporting the on-target efficacy of the inhibitor. Encouraged by the therapeutic efficacy of 4-MU in PCa cell lines, we expanded our investigation into clinical prostate tumours using a PDE model [35]. Treatment of PCa PDE tissues with 4-MU for 48 h resulted in marked suppression of the proliferative marker Ki67, from a mean of 26.2% positively stained epithelial cells in control-treated PDE tissues to 5.4% and 1.5% in PDE tissues treated with 0.5 mM and 1 mM 4-MU, respectively (Fig. 3j). This finding was validated in an independent cohort of PCa PDEs, where 0.5 mM and 1 mM 4-MU again significantly reduced Ki67 positivity compared to the control (Supplementary Figure 2l). In summary, HMMR expression is critical for PCa cell growth and survival and represents a potential therapeutic target.

### Targeting HMMR inhibits AR nuclear localisation and transcriptional activity

**Fig. 4:**
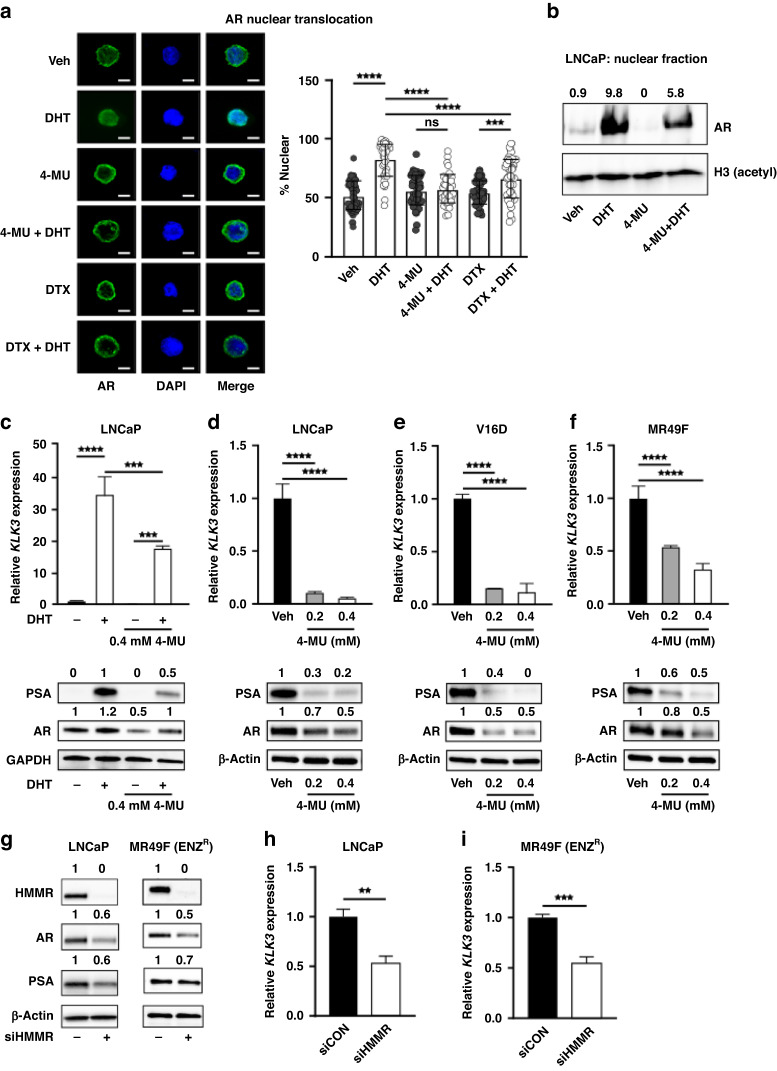
HMMR inhibition decreases AR nuclear translocation and transcriptional activity. **a** Decreased nuclear localisation of pCMV-tagged wtAR (green) in transfected PC3 cells treated with 4-MU or docetaxel (DTX) ± dihydrotestosterone (DHT), captured by fluorescence microscopy. Nuclei were visualised with DAPI mounting media (blue). Data are presented as the mean ± SD of 2 wells and represent two independent experiments. Data were analysed using two-way ANOVA with Tukey’s multiple comparison test. (***p < 0.001, ****p < 0.0001). The scale bar represents 10 pixels/micron. **b** Western Blot of AR protein expression in LNCaP nuclear fraction upon treatment with vehicle, 10 nM DHT, 0.4 mM 4-MU or DHT + 4-MU. Acetylated Histone H3 was used as a nuclear marker. Numerals above each lane represent densitometric analysis of AR relative to H3. **c** LNCaP cells were androgen deprived for 3 days prior to treatment with DHT ± 4-MU. Cells were analysed by RT-qPCR (upper panel) or Western Blot (lower panel) for KLK3 expression. Genes were normalised to *GUSB* and *L19*. Loading control for Western Blot was GAPDH. PCa cells grown in the presence of androgens were treated with increasing doses of 4-MU. Cells were analysed by RT-qPCR (upper panel) or Western Blot (lower panel) for KLK3 expression in LNCaP (**d**), V16D (**e**) and MR49F (**f**) cells. Genes were normalised to GUSB and L19. Loading control for Western Blots was β-Actin. Numerals above each lane represent densitometric analysis of each protein relative to β-Actin. Data in **c**–**f** are presented as mean ± SD of 3 wells and analysed using one-way ANOVA with Dunnett’s test (***p < 0.001, ****p < 0.0001). **g** HMMR, AR and PSA protein expression after 72 h of siHMMR knockdown. Numerals above each lane represent densitometric analysis of each protein relative to the loading control β-Actin. Data represents two independent experiments. RT-qPCR of *KLK3* expression in response to 72 h of siHMMR knockdown in LNCaP (**g**) and MR49F (**h**) cells. Genes were normalised to *GUSB* and *L19*. Data are presented as mean ± SD of three biological replicates and two independent experiments. siControl was set to one and data was analysed by unpaired student’s T-test (siHMMR vs siCON; **p < 0.01, ***p < 0.001).

HMMR is a protein with dual functions: extracellularly, HMMR is a receptor for hyaluronic acid; intracellularly, HMMR associates with microtubules to regulate protein trafficking and spindle assembly prior to mitosis [36]. In PCa, the microtubule network is particularly significant as it directly facilitates AR nuclear translocation and transcriptional activation of downstream AR signalling [37, 38]. In fact, the reliance of AR on microtubules is attributed to the success of microtubule targeting agent docetaxel in PCa, which is the first-line treatment and standard-of-care for metastatic CRPC [39, 40]. To determine whether the microtubule aspect of HMMR underlies its role in PCa cell growth and survival, we investigated whether HMMR inhibition alters the cellular localisation of AR. Exogenous wild-type AR was transiently transfected into AR-negative PC3 PCa cells and treated with vehicle control or DHT in the presence or absence of 4-MU. As expected, AR was predominantly localised in the nucleus upon DHT treatment (83.19%) compared to control (51.74%) (Fig. 4a). Treatment with 4-MU alone (56.11%) did not induce AR localisation, and the presence of 4-MU prevented the nuclear import of AR upon DHT treatment (57.56%) (Fig. 4a), to a similar extent as the microtubule inhibitor docetaxel in the presence of DHT (66.63%) (Fig. 4a). We further showed by subcellular fractionation that co-treatment with 4-MU and DHT reduces nuclear localisation of AR in LNCaP cells compared to DHT treatment alone (Fig. 4b, Supplementary Figure 3a). We next evaluated PSA expression in LNCaP cells as a downstream readout of AR nuclear transcriptional activity, and found that 4-MU reduced DHT-mediated induction of PSA at both the gene and protein levels (Fig. 4c). Interestingly, we observed a decrease in AR steady-state protein levels with 4-MU treatment alone, which was stabilised by addition of DHT (Fig. 4c). In LNCaP, V16D and MR49F cells cultured in growth medium containing full serum, to represent physiological levels of androgens, 4-MU dose-dependently reduced expression of PSA at both the gene and protein levels (Fig. 4d–f). The effect of 4-MU on AR protein expression was again observed and most prominently in V16D Cells (Fig. 4d–f). To validate the effects of HMMR inhibition by 4-MU on AR signalling, we examined AR and PSA levels upon HMMR knockdown. Depletion of HMMR with siRNA reduced AR protein levels by up to 50% (Fig. 4g), and PSA gene and protein levels were subsequently decreased, in both LNCaP and MR49F cells (Fig. 4g–i). Collectively, these findings reveal that targeting HMMR results in the inhibition of AR signalling, mediated through decreased AR nuclear translocation and expression.

### Targeting HMMR enhances enzalutamide efficacy

**Fig. 5:**
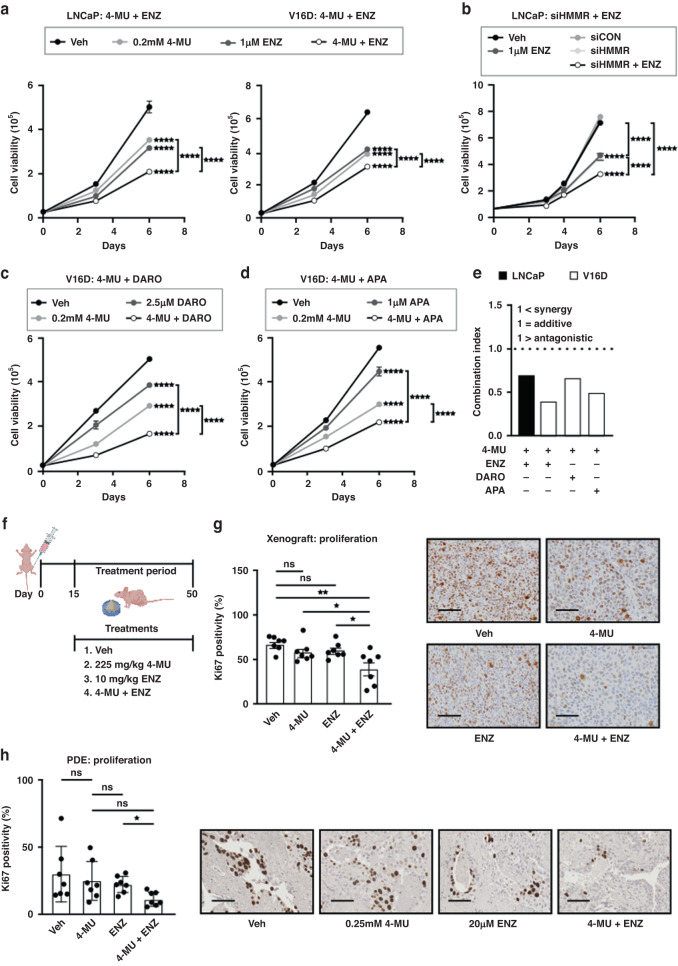
Combining HMMR inhibition with ARSI treatment enhances suppression of prostate cell proliferation and tumour growth. **a** Combination treatment with 4-MU + ENZ significantly inhibits LNCaP and V16D PCa cell viability compared to Vehicle, 4-MU or ENZ treatment alone. Cells were counted on days 3 and 6 post-treatment using Trypan blue dye exclusion. Data are presented as mean ± SD of triplicate wells, represent two independent experiments, and were analysed using two-way ANOVA with Tukey’s multiple comparison test (****p < 0.0001). **b** Combination treatment with siHMMR+ENZ significantly inhibits LNCaP PCa cell viability compared to Vehicle, 4-MU or ENZ treatment alone, as assessed by Trypan blue dye exclusion. Data are presented as mean ± SD of triplicate wells, represent two independent experiments, and were analysed using two-way ANOVA with Tukey’s multiple comparison test (****p < 0.0001). **c**, **d** Combination treatment with 4-MU+darolutamide (DARO) or 4-MU+apalutamide (APA) significantly inhibits V16D PCa cell viability compared to Vehicle, 4-MU or ENZ treatment alone, as determined by the Trypan blue dye exclusion. Data are presented as mean ± SD of triplicate wells, represent two independent experiments, and were analysed using two-way ANOVA with Tukey’s multiple comparison test (****p < 0.0001). **e** The Chou-Talalay method [17] was used to determine the combination indices of the growth curves (A, B, D, E). **f** Schematic diagram of treatment regimen for in vivo subcutaneous xenograft model. **g** Combination treatment of 4-MU + ENZ significantly inhibits the proliferation of V16D PCa xenograft tumours compared to 4-MU or ENZ treatment alone. At the completion of the in vivo experiment, xenograft tumours were analysed for the proliferative marker Ki67 by immunohistochemistry (IHC). Quantification of Ki67 staining on the left and representative IHC images on the right. Data are presented as mean ± SD of 7 mice in each treatment group and analysed using one-way ANOVA with Turkey’s multiple comparison test (*p < 0.05, **p < 0.01). **h** Combination treatment with 4-MU + ENZ inhibits proliferation in patient-derived prostate cancer explants (PDEs). PDEs (*n* = 7) were treated as indicated for 48 h, then paraffin-embedded and formalin-fixed prior to immunohistochemistry (IHC) with proliferation marker Ki67. Digital images were manually counted. Quantification of Ki67 staining on the left and representative IHC images on the right. Data are presented as mean ± SD of 7 patients and analysed using one-way ANOVA with Dunnett’s multiple comparison test (*p < 0.05).

The observations that (1) *HMMR* is not modulated by enzalutamide, and (2) HMMR inhibition prevents AR transcriptional activity, provide a rationale for combining HMMR-targeted therapies with ARSIs as a potential new therapeutic strategy. Treatment of LNCaP and V16D PCa cells with enzalutamide in the presence or absence of 4-MU revealed that 4-MU enhances the efficacy of enzalutamide (Fig. 5a). Similarly, co-treatment with enzalutamide and siHMMR resulted in significant suppression of LNCaP viability compared to enzalutamide treatment or siHMMR treatment alone (Fig. 5b). Co-administration of 4-MU with darolutamide (Fig. 5c) or apalutamide (Fig. 5d) also significantly reduced cell viability compared to treatment with the individual agents. The Combination Index (CI) for each ARSI with 4-MU was calculated, which is a measure for determining whether a drug combination effect is synergistic (CI < 1), antagonistic (CI > 1) or additive (CI = 1) [17]. In both LNCaP and V16D cells, the CI for each combination was <1, indicative of synergy between 4-MU and ARSIs (Fig. 5e). To evaluate the combination of 4-MU and enzalutamide in vivo, we employed the V16D tumour xenograft model [41] according to the experimental plan depicted in Fig. 5f. Co-administration of 4-MU and enzalutamide markedly reduced tumour cell proliferation, as determined by immunostaining for the proliferative marker Ki67 (Fig. 5g). Tumour size was also significantly reduced by the combination of 4-MU and enzalutamide when compared to vehicle or enzalutamide treatment alone, but not compared to 4-MU single treatment (Supplementary Fig. 4a). An improvement in survival with the combination of 4-MU and enzalutamide was observed compared to the vehicle or single treatment groups (Supplementary Fig. 4b). Serum PSA levels remained unchanged across all treatments (Supplementary Figure 4c). Body weight measurements taken over the course of the study suggest no adverse effects of any treatment (Supplementary Figure 4d). Evaluation of the combination in PCa PDEs showed a significant reduction in epithelial cell proliferation from 29.42% Ki67 positively stained cells in control-treated PDE tissues to 11.01% upon co-treatment with 4-MU and enzalutamide (Fig. 5h). Compared to each agent when used individually, the combination was not significantly different to 4-MU treatment alone (24.51% Ki67 positively stained cells), but significantly suppressed proliferation compared to enzalutamide alone (21.91% ki67 positively stained cells) (Fig. 5h). Our results demonstrate in multiple models of PCa, including cell line and xenograft models of CRPC, and in clinically derived prostate tumours, that targeting HMMR enhances the anti-proliferative efficacy of enzalutamide.

## Discussion

ARSIs have revolutionised the treatment of advanced PCa but are unable to achieve durable responses, highlighting that combination approaches may be needed to overcome resistance. To rationally design an effective combination strategy, we used a data-driven approach wherein AR knockdown with siRNA was used as the benchmark of AR inhibition in PCa cells. *HMMR* was identified as an AR-regulated gene that is not suppressed by ARSIs enzalutamide, apalutamide or darolutamide. This study reveals HMMR as a survival factor in PCa and a promising co-target worth exploring to overcome resistance to ARSIs in PCa.

The potential role of *HMMR* in cancer development and progression is linked to two main functions, namely HA-induced cell migration and cell cycle progression [42–44]. *HMMR* overexpressing cells exhibit increased migratory and proliferative potential and the converse is true for *HMMR* downregulated cells [29, 45]. We observed a significant reduction in cell proliferation in a range of PCa cell lines following HMMR knockdown. Previous studies support our findings in enzalutamide-sensitive LNCaP cells [27, 46], whereas we provide the first-in-field evidence that HMMR is a potential target in CPRC, and importantly, in a setting of enzalutamide resistance. Given that there are no commercially available HMMR inhibitors, we employed a hyaluronic acid synthesis inhibitor 4-MU to suppress downstream HMMR activity [34, 47]. Hyaluronic acid is a glycosaminoglycan that forms an integral part of the extracellular matrix and has been implicated in the development and progression of PCa [47, 48]. Of note, high HA expression in the tumour stroma of PCa tissues is associated with increased proliferation, metastasis and disease recurrence post-radical prostatectomy [49–52]. The tumour-promoting roles of HA are linked to its interaction with two main receptors, CD44 and HMMR (RHAMM) [47]. Whilst both receptors have been detected in PCa cells, CD44 is reported to be highly expressed in androgen-independent and/or AR-negative cells whilst HMMR (RHAMM) is expressed in all PCa cell types [53–55]. Earlier studies also clearly show that CD44 expression does not correlate with poorer patient outcomes, PSA recurrence or metastasis in PCa patients [27, 49]. These observations suggest, however, that HMMR may be the predominant receptor-promoting HA-mediated oncogenic phenotypes in PCa. Indeed, we observed that treatment with 4-MU recapitulated mechanistic changes similar to HMMR knockdown cells.

Consistent with the microtubule-associated functions of HMMR [37], we saw a decrease in AR nuclear localisation with 4-MU treatment post DHT stimulation that resulted in decreased expression of well-known AR-regulated genes, and of AR itself. While it is possible that the localisation effects were due to the decreased AR steady-state levels observed upon 4-MU treatment, AR expression was not completely lost at the doses used in this study, and the AR that was expressed was clearly being held out of the nucleus in the presence of 4-MU. We propose that 4-MU instead works in a similar way to docetaxel, which is known to reduce AR expression by blocking AR nuclear localisation [56]. How this occurs mechanistically has not been defined for docetaxel, but studies into the regulation of AR by tumour suppressor PTEN may provide some insight. PTEN is known to suppress AR nuclear localisation, and the retention of AR in the cytoplasm promotes AR degradation via enzymatic factors [57].

In summary, as the AR continues to evolve in response to monotherapy, it is becoming more apparent that combination treatments may be critical for improving PCa survival. The work presented in this study highlights the importance of maximising the suppression of AR signalling using combinatorial approaches to achieve better control of cancer growth. We present strong evidence that persistent HMMR expression despite treatment with ARSIs provides a survival benefit to PCa cells and that co-targeting both HMMR and AR has promising therapeutic implications for the management of advanced PCa.

### Supplementary information

Supplementary Data Files

Essential 10 ARRIVE guidelines

## Data Availability

1. Gillis, J. L., J. A. Hinneh, N. K. Ryan, S. Irani, M. Moldovan, L.-E. Quek, R. K. Shrestha, A. R. Hanson, J. Xie, A. J. Hoy, J. Holst, M. M. Centenera, I. G. Mills, D. J. Lynn, L. A. Selth and L. M. Butler (2021). **NCBI Gene Expression Omnibus: GSE152254** “A feedback loop between the androgen receptor and 6-phosphogluoconate dehydrogenase (6PGD) drives prostate cancer growth.” eLife 10: e62592. https://www.ncbi.nlm.nih.gov/geo/query/acc.cgi?acc=GSE152254 2. Gonit, M, Zhang, J, Salazar, MdA, Cui, H, Shatnawi, A, Trumbly, R & Ratnam, M 2011. **NCBI Gene Expression Omnibus:** GSE22483 ‘Hormone Depletion-Insensitivity of Prostate Cancer Cells Is Supported by the AR Without Binding to Classical Response Elements’, *Molecular Endocrinology*, vol. 25, no. 4, pp. 621-634. https://www.ncbi.nlm.nih.gov/geo/query/acc.cgi?acc=GSE22483 3. Iglesias-Gato DThysell ETyanova SCrnalic SSantos ALima TSGeiger TCox JWidmark ABergh AMann MFlores-Morales AWikström P (2018). ProteomeXchange ID PXD009868. The Proteome of Prostate Cancer Bone Metastasis Reveals Heterogeneity with Prognostic Implications. https://clincancerres.aacrjournals.org/content/24 4. TCGA (2019) National Cancer Institute ID TCGA-PRAD. The Cancer Genome Atlas Prostate Adenocarcinoma (TCGA-PRAD). https://portal.gdc.cancer.gov/projects/TCGA-PRAD 5. Taylor BS, Schultz N, Hieronymus H, Gopalan A, Xiao Y, Carver BS, Arora VK, Kaushik P, Cerami E, Reva B, Antipin Y, Mitsiades N, Landers T, Dolgalev I, Major JE, Wilson M, Socci ND, Lash AE, Heguy A, Eastham JA, Scher HI, Reuter VE, Scardino PT, Sander CSawyers CL, Gerald WL (2010) **NCBI Gene Expression Omnibus** ID GSE21032.
