# Supplementary material for: Targeting hyaluronan-mediated motility receptor (HMMR) enhances response to androgen receptor signalling inhibitors in prostate cancer
Source: Br J Cancer. 2023 Sep 6;129(8):1350–61. doi: 10.1038/s41416-023-02406-8 (PMC10575850; doi:10.1038/s41416-023-02406-8)
Supplement: Supplementary file 1 — Supplementary Data Files [file 41416_2023_2406_MOESM1_ESM.docx]

**Supplementary Figure 1**

**
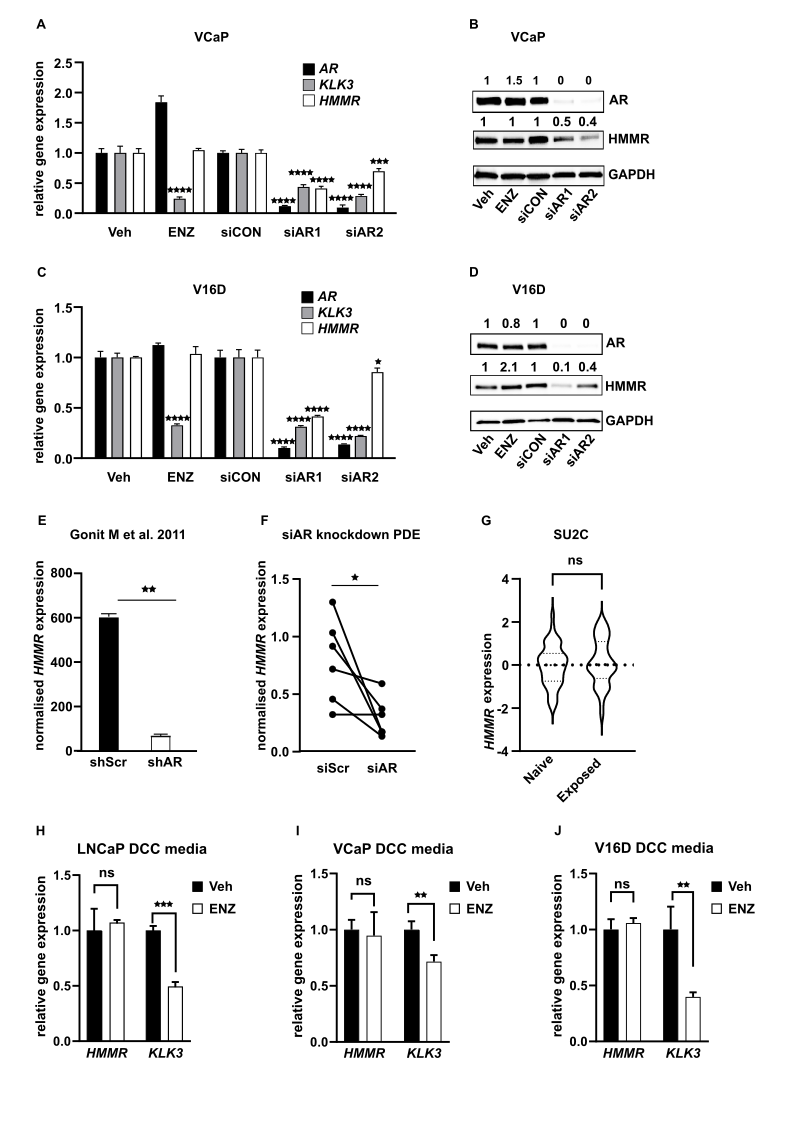
**

**Figure legend for Supplementary Figure 1**

(**A-D**) Validation of HMMR expression in response to siAR or 1µM ENZ by RT-qPCR or Western Blot in VCaP (A-B) or V16D (C-D) PCa cells. All RT-qPCR data were normalised to *GUSB* and *L19*. Data are presented as mean ± SD of 3 biological replicates and represent two independent experiments. Controls (Veh and siCON) were set to one and statistically evaluated using two-way ANOVA with Tukey’s multiple comparison test (treatment vs control; *p<0.05, ***p<0.001, ****p<0.0001). For Western Blots, GAPDH was used as loading control. Numerals above each lane represent densitometric analysis of each protein relative to loading controls GAPDH. (**E**) RNAseq data from Gonit et al confirm HMMR downregulation in AR stable knockdown PCa cells. Data were analysed using unpaired student’s T-test (**p<0.01,). (**F**) RT-qPCR showing HMMR mRNA expression in six AR depleted PDEs using siRNA-loaded nanoparticles. Data were analysed using unpaired student’s T-test (*p<0.05). (**G)** HMMR expression remains unchanged in SU2C CRPC patients treated with ARSI (exposed) compared to untreated patients (naïve). (**H-J**) LNCaP, VCaP and V16D cells were cultured in androgen-deprived conditions for three days and then treated with vehicle or 1 µM ENZ for 24 h. RT-qPCR showing *HMMR* and *KLK3* expression in LNCaP (H), VCaP (I) and V16D (J). Genes were normalised to *GUSB* and *L19*. Vehicle control was set to one and analysed with unpaired student T-test (**p<0.01, ***p<0.001).

**Supplementary Figure 2**

**
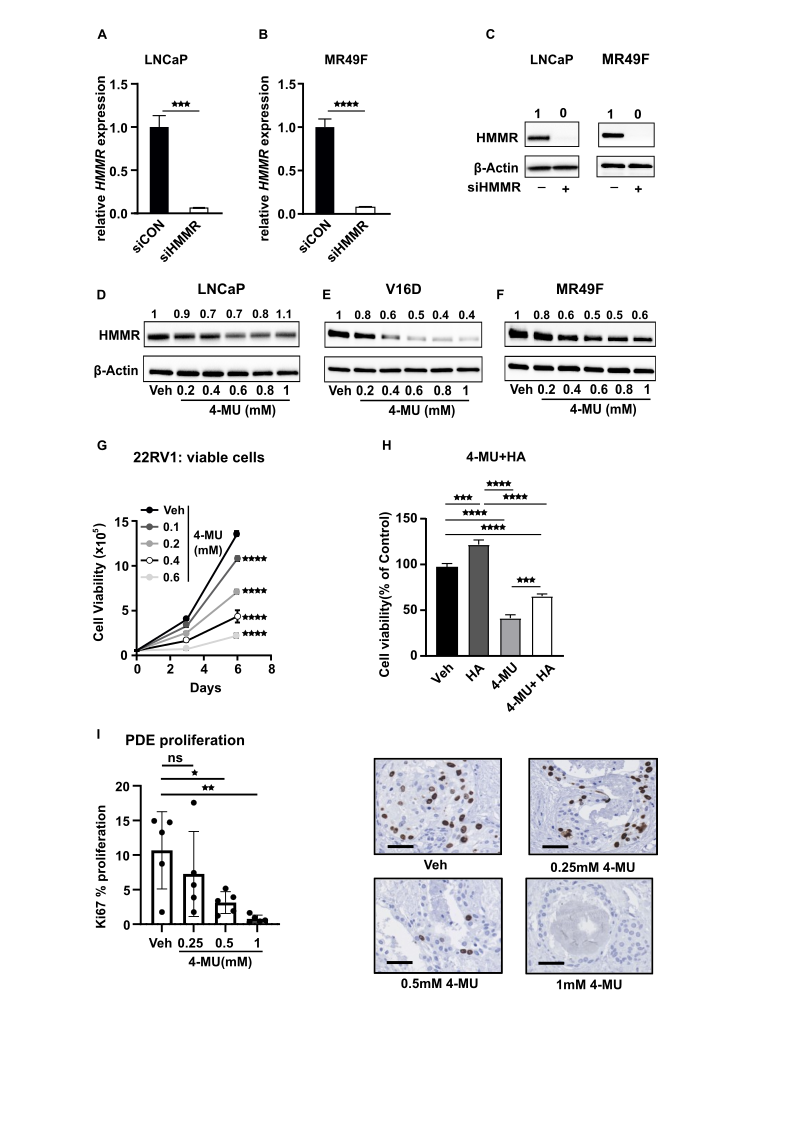
**

**Figure legend for Supplementary Figure 2**

(**A-B**) *HMMR* mRNA expression in LNCaP (A) and MR49F (B) after 48 h of *HMMR* knockdown with siHMMR. Gene expression was normalised to *GUSB* and L19. Data are presented as mean ± SD of 3 biological replicates, represent two independent experiments, and were analysed by unpaired student’s T-test (***p<0.001, ****p<0.0001). (**C**) HMMR protein expression in LNCaP and MR49F PCa cells after 48 h of HMMR knockdown by siHMMR. β-Actin was used as loading control. (**D-F**) HMMR protein expression in response to 4-MU treatment in LNCaP (D), V16D (E), or MR49F (F) PCa cells after 24 h of treatment. β-Actin was used as loading control. Numerals above each lane represent densitometric analysis of each protein relative to loading controls β-Actin. (**G**) 4-MU inhibits 22RV1 PCa cell viability dose-dependently as determined by Trypan blue dye exclusion. Cells were counted manually after -3 and -6 days of treatment. Data are mean ± SD of triplicate wells, represent two independent experiments, and were analysed using two-way ANOVA with Tukey’s multiple comparison test (****p<0.0001). (**H**) Hyaluronic Acid (HA) partially rescued the inhibitory effect of 4-MU on V16D PCa cells. Cell viability was determined by Trypan Blue dye exclusion following 6 days of treatment with vehicle, 0.4 mM 4-MU, 50 µg/ml HA, or 4-MU+HA. Data presented as mean ± SD of triplicate wells and was statistically analysed using one-way ANOVA with Tukey’s multiple comparison test (treatments *vs* control; ***p<0.001, ****p<0.0001). Data represents two independent experiments. (**I**) 4-MU inhibits prostate cancer cell proliferation in patient-derived explants (PDEs). PDEs (n=5) were treated with increasing dose of 4-MU for 48 h, then paraffin-embedded and formalin-fixed prior to immunohistochemistry analysis with proliferative marker Ki67. Positive and negatively stained cells were manually counted and proliferation calculated as percent of positively stained cells out of the total cells counts. Data presented as mean ± SEM and were statistically analysed using one-way ANOVA with Dunnett’s test (*p<0.05, **p<0.01). Quantification of Ki67 staining on the left and representative IHC images on the right.

**Supplementary Figure 3**

**
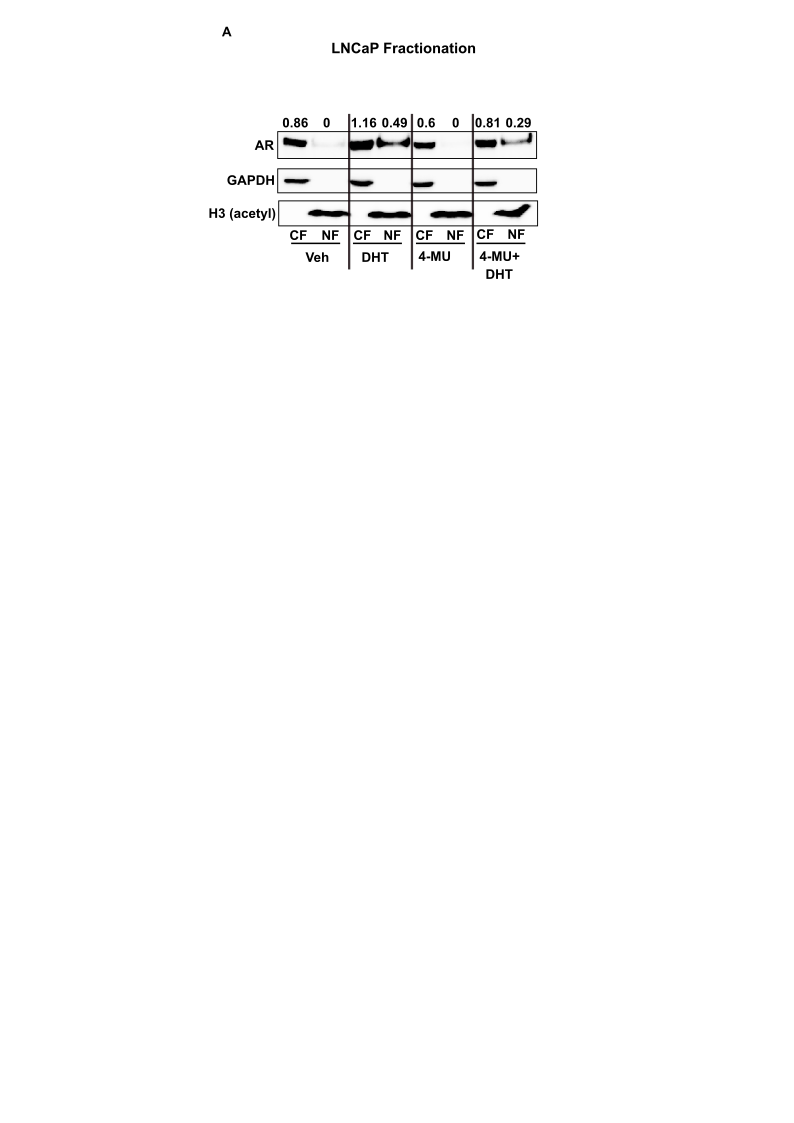
**

**Figure legend for Supplementary Figure 3**

Western Blot of AR expression in the cytoplasmic fraction (CF) or nuclear fraction (NF) of LNCaP cells following treatment with vehicle, 10 nM DHT, 0.4 mM 4-MU, or DHT+4-MU. GAPDH was used as the cytoplasmic marker and acetylated histone H3 as the nuclear marker. Numerals above each lane represent densitometric analysis of AR, relative to GAPDH for the CF and H3 for the NF.

**Supplementary Figure 4**

**
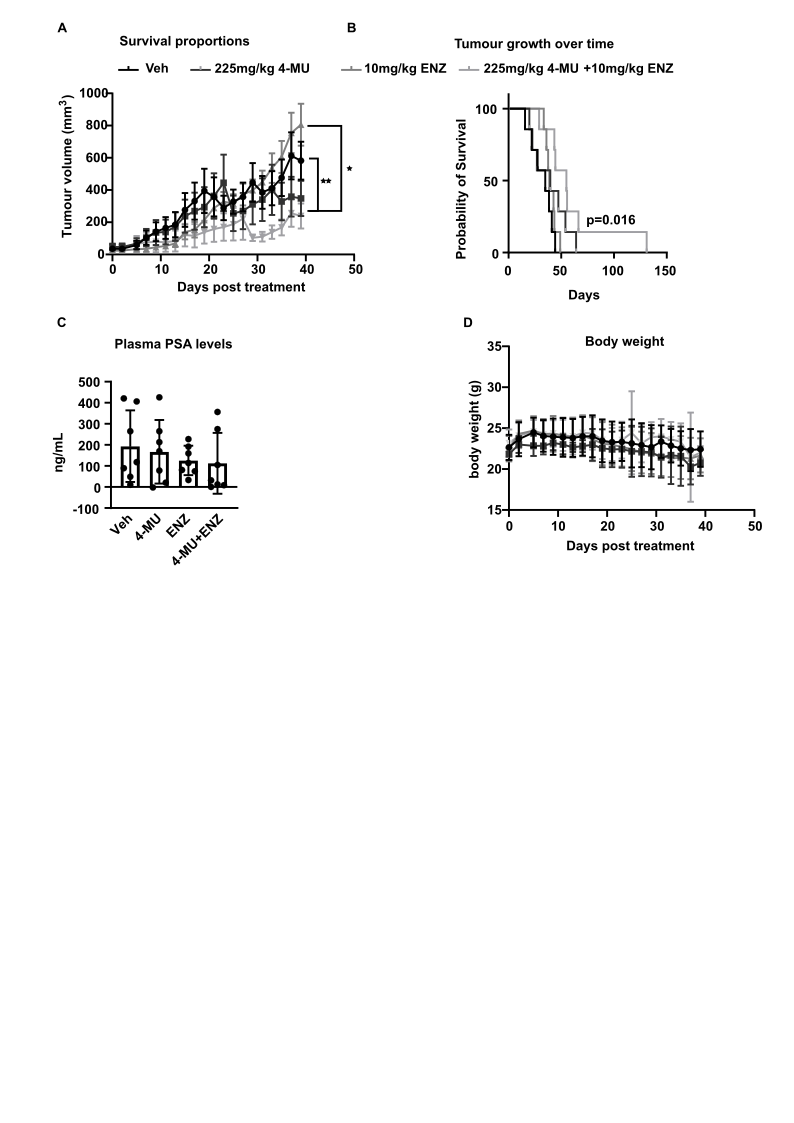
**

**Figure legend for Supplementary Figure 4**

**(A)** Tumour volume in mice treated for 5 weeks with vehicle, 4-MU, ENZ or 4-MU+ENZ (n=7 per treatment). Data were analysed with one-way ANOVA with Tukey’s multiple comparison test and are representative of the mean ± SD of 7 mice (treatment *vs* control; *p<0.05, **p<0.01). **(B)** Time to tumour volume endpoint of 1000 mm^3^ was calculated with the formula = [log10 (endpoint tumour volume-c)/m]; where c is the y-intercept and m is the slope of the straight line obtained from the linear regression of the log-transformed tumour growth data. The time to tumour endpoint, expressed in days was determined for each mouse and the survival curve derived with the aid of a Kaplan-Meier analysis. Log-rank Mantel-Cox test was used to determine significance between groups. **(C)** Plasma samples from the vehicle, single agents or combination treated groups were analysed for PSA levels by ELISA. Data were analysed using one-way ANOVA with Tukey’s multiple comparison test and are representative of the mean ± SD of 7 mice per group. **(D)** Body weight of mice taken at regular intervals over 5 weeks of treatment with vehicle, 4-MU, ENZ or 4-MU+ENZ. Data was analysed using one-way ANOVA with Tukey’s multiple comparison test.

**Supplementary Table 1 Primer sequences**

| Reagents type (species or source) | Designation | Source or reference | identifier | Additional information |
| --- | --- | --- | --- | --- |
| Sequenced-based reagent | AR-F | This paper | qRT-PCR primers | CAACTCCTTCAGCAACAGCA |
| Sequenced-based reagent | AR-R | This paper | qRT-PCR primers | TCGAAGTGCCCCCTAAGTAA |
| Sequenced-based reagent | FKBP5-F | This paper | qRT-PCR primers | AAAAGGCCAAGGAGCACAAC |
| Sequenced-based reagent | FKBP5-R | This paper | qRT-PCR primers | TTGAGGAGGGGCCGAGTTC |
| Sequenced-based reagent | GUSB-F | This paper | qRT-PCR primers | CGTCCCACCTAGAATCTGCT |
| Sequenced-based reagent | GUSB-R | This paper | qRT-PCR primers | TTGCTCACAAAGGTCACAGG |
| Sequenced-based reagent | HMMR-F | This paper | qRT-PCR primers | AAGCTGACAGCGGAGTTTTG |
| Sequenced-based reagent | HMMR-R | This paper | qRT-PCR primers | TGCTAAATTATTGGGTCATCAGAA |
| Sequenced-based reagent | KLK2-F | This paper | qRT-PCR primers | GGTGGCTGTGTACAGTCATGGAT |
| Sequenced-based reagent | KLK2-R | This paper | qRT-PCR primers | TGTCTTCAGGCTCAAACAGGTTG |
| Sequenced-based reagent | KLK3(PSA)-F | This paper | qRT-PCR primers | ACCAGAGGAGTTCTTGACCCCAAA |
| Sequenced-based reagent | KLK3(PSA)-R | This paper | qRT-PCR primers | CCCCAGAATCACCCGAGCAG |
| Sequenced-based reagent | L19-F | This paper | qRT-PCR primers | TGCCAGTGGAAAAATCAGCCA |
| Sequenced-based reagent | L19-R | This paper | qRT-PCR primers | CAAAGCAAATCTCGACACCTTG |
| Sequenced-based reagent | TMPRSS2-F | This paper | qRT-PCR primers | GACCAAGAACAATGACATTGCG |
| Sequenced-based reagent | TMPRSS2-R | This paper | qRT-PCR primers | GTTCTGGCTGCAGCATCATG |

**Supplementary Table 2 Antibodies**

| Antigen | Source | Identifier | Additional information |
| --- | --- | --- | --- |
| AR-N20 (rabbit polyclonal) | Santa Cruz Biotechnology Inc | sc-816 | Western blot (1:1000) |
| GAPDH (hFAB Rhodamine) | BioRad | 12004167 | Western blot (1:1000) |
| HMMR (rabbit monoclonal to CD168) | Abcam | ab108339, RRID:AB_10861654 | Western blot (1:1000) |
| acetyl-Histone H3 (rabbit polyclonal) | Millipore, Sigma Aldrich | 06-599, 631270 | Western blot (1:1000) |
| PSA (rabbit polyclonal) | ProteinTech Group Inc. | 10679-1-AP | Western blot (1:1000) |
| β-Actin (AC-15) (mouse monoclonal) | Sigma Aldrich | A5441 | Western blot (1:1000) |
| Anti-human Ki67 (MIB1) (mouse monoclonal) | DAKO, Agilent technologies | M7240 | Immunohistochemistry on PDE tissues (1:200) |
| Ki67 (mouse monoclonal) | Agilent Technologies | M724001-2 | Immunohistochemistry on animal tissues (1:200) |
