# Supplementary material for: Targeting hyaluronan-mediated motility receptor (HMMR) enhances response to androgen receptor signalling inhibitors in prostate cancer
Source: Br J Cancer. 2023 Sep 6;129(8):1350–61. doi: 10.1038/s41416-023-02406-8 (PMC10575850; doi:10.1038/s41416-023-02406-8)
Supplement: Supplementary file 2 — Essential 10 ARRIVE guidelines [file 41416_2023_2406_MOESM2_ESM.docx]

**ARRIVE Guidelines Essential 10**

| Study design | Balb/ cSlc (*nu/nu*) 6 weeks old male nude mice were subcutaneously injected with 1:1 PBS/matrigel suspension containing 2x10^6^ V16D cells. Once tumours became palpable, mice were assigned randomly to one of the 4 groups (Vehicle, 225 mg/kg 4-MU, 10 mg/kg ENZ and 225 mg/kg 4-MU+10 mg/kg ENZ). For 5 weeks, mice were fed with powdered diet supplemented with vehicle or 4-MU, ENZ or 4-MU+ENZ. |
| --- | --- |
| Sample size | There were 10 mice per treatment group. In total 40 mice were used for the study.  Sample size was calculated to detect effects at the 5% alpha level with 80% power. Based on our previous research with these models, and literature values, it was determined that in order to detect a treatment-related difference in tumour volume of 30% and assuming a coefficient of variation of 0.15, a sample of 8  mice per group would be needed to detect this effect at the requisite power. We requested 10 mice per group to account for the variation in tumour take rate and unexpected. |
| Inclusion and exclusion criteria | Mice who did not develop tumours were excluded from the study. |
| Randomisation | Tumour-bearing mice were randomly distributed into unlabelled cages by two neutral people who were unaware of the treatment groups. Cages were then labelled in no particular order to receive different treatments. |
| Blinding | Blinding was carried out at the randomisation or allocation stage. The treatment period did not require blinding. |
| Outcomes | The following parameters were assessed: tumour volume and body weight were measured every second day. At the endpoint of the study, tumour tissues were collected and stained for Ki67, a marker of proliferation. Time to endpoint tumour volume was extrapolated and a Kaplan-Meier curve was generated to determine the effect of treatment on survival. |
| Statistical method | Comparison between groups was achieved using one-way ANOVA with Tukey’s multiple comparison test on GraphPad Prism 9.0. A p value of ≤ 0.05 was considered statistically significant. |
| Experimental animals | Mus musculus,  Balb/ cSlc (*nu/nu*) NOD SCID,  5-6 weeks male mice |
| Experimental procedure | 2x10^6^ cells/100 µl of V16D cell suspension were injected into the lower right flank of each mouse under anaesthesia using isoflurane. When tumours became palpable, mice were randomly assigned into one of four groups (Vehicle, 225 mg/kg 4-MU, 10 mg/kg ENZ and 225 mg/kg 4-MU+10 mg/kg ENZ). Food consumption was measured using powdered diet delivered via powder feeder bowls. Initial food consumption was 4 g/ day for each mouse. The required amount of 4-MU and ENZ was dissolved in DMSO to obtain a stock concentration of 500 mg/mL and 100 mg/mL, respectively. The amount of drug needed per group was calculated based on the average body weight of each group. The required volume of drug was dusted over the powdered food and rigorously mixed for 10 min by hand, followed by 5 min mixing using a mortar and pestle to ensure even distribution. Daily food intake was measured, and drug concentration adjusted every other day according to body weight changes. Mice were treated for a period of 5 weeks.  Mice with tumour volume of 1000 mm^3^, body weight below 20% of the weight at the start of the experiment, or outward signs of severe illness were culled before the end of the study. The remaining mice were culled at the end of 5 weeks of treatment. Mice were anaesthetised with isoflurane at a rate of 2.5% and maintained at 1.8% for tumour resection. The resected tumours were weighed and tumour volume was measured. The tumour was formalin fixed for immunohistochemistry (IHC) analysis. Mice were culled and blood samples collected by cardiac puncture into heparinised tubes and centrifuged at 2500 rpm for 10 min to obtain plasma, which was used for assessing PSA levels. |
| Results | Combination of 4-MU + ENZ significantly decreased proliferation of tumour cells compared to the vehicle or individual treatments (Data are presented as mean ± SD of 7 mice in each group and analysed using one-way ANOVA with Turkey’s multiple comparison test). The time to tumour endpoint, expressed in days was determined for each mouse and our results showed that 4-MU + ENZ improved the survival of mice compared to the individual or vehicle treated groups. The survival curve was derived with the aid of a Kaplan-Meier analysis. Log-rank Mantel-Cox test was used to determine the significance between the groups.  Plasma samples from the vehicle, single agents or combination treated groups were analysed for PSA levels by ELISA. There were no significant changes in plasma PSA levels irrespective of treatment. Data were analysed using one-way ANOVA with Tukey’s multiple comparison test and are representative of the mean ± SD of 7 mice per group. |
